# How do they learn: types and characteristics of medical and healthcare student engagement in a simulation-based learning environment

**DOI:** 10.1186/s12909-021-02858-7

**Published:** 2021-08-06

**Authors:** Yashuang Wang, Yan Ji

**Affiliations:** 1 Institute of Education, Nanjing University, Nanjing, 210093 China; 2 School of Nursing, Nanjing Medical University, Nanjing, 211166 China

**Keywords:** Simulation-based learning, Medical and healthcare students, Student engagement, Medical and healthcare education, Learning conducive environment, Facilitator-student learning relationships

## Abstract

**Background:**

Student engagement can predict successful learning outcomes and academic development. The expansion of simulation-based medical and healthcare education creates challenges for educators, as they must help students engage in a simulation-based learning environment. This research provides a reference for facilitators of simulation teaching and student learning in medical and health-related majors by providing a deep understanding of student engagement in a simulation-based learning environment.

**Methods:**

We conducted semi-structured interviews with ten medical and healthcare students to explore their learning types and characteristics in a simulation-based learning environment. Thematic analysis was used to analyse the data.

**Results:**

The interviews were thematically analysed to identify three types of student engagement in the simulation-based learning environment: reflective engagement, performance engagement, and interactive engagement. The analysis also identified eight sub-themes: active, persistent, and focused thinking engagement; self-directed-learning thinking engagement with the purpose of problem solving; active “voice” in class; strong emotional experience and disclosure; demonstration of professional leadership; interaction with realistic learning situations; support from teammates; and collegial facilitator-student interaction.

**Conclusions:**

The student interview and thematic analysis methods can be used to study the richness of student engagement in simulation-based learning environments. This study finds that student engagement in a simulation-based learning environment is different from that in a traditional environment, as it places greater emphasis on performance engagement, which combines both thinking and physical engagement, as well as on interactive engagement as generated through interpersonal interactions. Therefore, we suggest expanding the learning space centring around “inquiry”, as it can help strengthen reflective communication and dialogue. It also facilitates imagination, stimulates empathy, and builds an interprofessional learning community. In this way, medical and healthcare students can learn through the two-way transmission of information and cultivate and reshape interpersonal relationships to improve engagement in a simulation-based learning environment.

**Supplementary Information:**

The online version contains supplementary material available at 10.1186/s12909-021-02858-7.

## Background

Student engagement is a key topic that has become very popular and widely researched in academia. Studies have shown that student engagement can directly predict learning outcomes [1], critical thinking skills [2], academic achievement and learning satisfaction [3]. Therefore, student engagement has become an important indicator of the quality of higher education. Countries such as the United States, China, the United Kingdom, and Australia have conducted national surveys of college student engagement [4]. College student engagement usually refers to the time and effort that college students invest in participating in activities with an educational purpose [5]. The classic “three-dimensional theory” of student engagement identifies behavioural engagement, cognitive engagement and affective engagement. Behavioural engagement emphasizes participation, which refers to students’ participation in academic, social and extracurricular activities; Cognitive engagement emphasizes investment, which refers to students’ willingness to invest effort to understand complex issues and master complex skills; emotional engagement refers to the positive or negative reactions of students to facilitators, peers, school, etc., and their emotional reactions in the classroom [6]. The “four-dimensional theory” includes academic, social, cognitive and affective engagement. Academic engagement includes behaviours related to direct participation in the learning process. Social engagement includes students’ behaviour in observing classroom discipline, lecturer-student interaction and peer interaction. Cognitive engagement refers to the deep thinking required to understand complex concepts. Affective engagement refers to the sense of identity and belonging to the school [7]. The main difference between the two theories is that the latter divides behavioural engagement into academic engagement and social engagement.

The diversity of learning fields gives rise to heterogeneity within the space and manners of medical and healthcare student engagement. Some scholars have studied medical and healthcare student engagement in the clinical environment by using the Moodle system, which is based on the visual learning environment. They found that students adopted a strategic learning approach in the clinical environment by using more Internet resources to answer medical questions under time constraints, in comparison to traditional learning techniques wherein students obtain information from books [8]. Medical and healthcare student engagement is also reflected in tasks that simulate clinical situations and online settings. Some scholars have studied the degree of medical student engagement in interprofessional cooperative communications and have researched the emotional obstacles to the e-learning engagement of medical students, including a sense of injustice, passivity and feeling overwhelmed [9, 10]. Increasingly, studies are conducting in-depth analyses of student engagement with different curricula and teaching styles. Researchers found that greater use of cognitive engagement, such as elaboration and critical thinking, is associated with higher levels of student performance in a medical gross anatomy course [11]. In addition, reflective writing exercises were found to be more likely to be an effective strategy than grades for fostering student engagement in medical humanities courses [12]. Research has also been conducted on the relationship between different learning styles and student engagement [13].

Medical and healthcare education is media- and resource-dependent in nature. It has become increasingly prevalent to integrate technology-enhanced learning (TEL) resources and promote the reform of information technology (IT)-based teaching models in higher medical and healthcare education [14]. The most representative example in the field is simulation-based medical and healthcare education, especially high-fidelity simulation, which can bridge the gap between theory and practice by immersing learners in a realistic setting [15]. In recent years, simulation-based training has seen increased popularity in global medical and healthcare education, and many discussions have been held on the inevitable reforms and future development trends [16]. The aim of such training is to improve the competence of medical and healthcare professionals, ensure the quality of medical and healthcare, and promote patient safety. Simulation-based education is conducted in a simulation-based learning environment. In contrast with traditional classrooms, the newly developed strategies and instructional methods(e.g., simulated clinical environment laboratory learning, virtual simulated equipment, team-based learning, problem-oriented learning, scenario, role-playing, virtual or standardised patients) profoundly change students’ learning environment and learning methods.

Driven by this movement, educators have begun to focus on medical and healthcare student engagement with the IT-based learning environment. Most research focuses on the effect of using IT in the objective physical environment and the impact of teaching methods on student engagement; such methods include classroom mobile technology [17], online learning aids [18], online tests [19], visualised virtual patients [20], audience response systems [21], simulation training [16, 22], etc. Many existing studies, including those listed above, focus on the impact of IT on student engagement, but little is known about what and how student engagement itself has changed. Medical and healthcare simulation-based training has changed traditional teaching and learning methods, and this has had a profound impact on the types and characteristics of student engagement. The simulation-based learning environment also poses challenges to the original classification and measurement of student engagement type within traditional classrooms, raising questions as to whether the original framework may still be applicable for the simulated environment. By studying medical and healthcare student engagement in the new learning environment and classifying the types and characteristics of student engagement, adjustments can be made to the teaching approaches that can help medical and healthcare students engage in meaningful learning in a more targeted manner.

At present, quantitative research is the most common research paradigm for studying college student engagement. However, many scholars have questioned the reliability and validity of the questionnaires using quantitative research and the validity of student responses [4, 23]. Researchers point out that student engagement is a complex, dynamic and contextualised concept that cannot be measured solely by quantitative surveys. Furthermore, students are prevented from expressing their thoughts beyond the questions within the questionnaire [24]. Therefore, current and future research methods should not be limited to large-scale questionnaires but extended to encompass multiple methods, including qualitative research because of its strength and suitability [25].

Medical and healthcare education has always been highly contextualised in a specific historical, ethnic, and regional development setting. The simulation-based learning environment involves various contextualised learning activities and practices that resemble real-life practice and experiences. The study of medical and healthcare student engagement in this environment needs to shift from the traditional quantitative research paradigm to a qualitative paradigm, as the latter can offer more insights into the interactive mechanisms between college students’ learning behaviour and the environment. Thus, the purpose of this study is to explore student perspectives following simulation-based learning to gain a more complete understanding of their engagement within the learning environment.

## Methods

### Interprofessional education course

The 10 participants all participated in an interprofessional education course that 48 students had taken as an optional course before the interview. The course has 10 teaching periods, each lasting 2 h. Seven periods were conducted in the discussion room, which was staffed by 1 tutor and 1 teaching assistant. Students were required to discuss cases in groups and reflect upon the professional value of their roles in the case. Students performed high-fidelity simulations in the laboratory twice accompanied by 3 tutors, one for “Neonatal respiratory distress syndrome rescue” and the other for “The rescue of cardiac and respiratory arrest patient with coronary heart disease”. Students in groups played different roles to run the case. Students made desktop deductions in the discussion room once under the guidance of 3 tutors, the theme of which was “The rescue of a large number of injured people suffering after a sudden explosion in the emergency room”. Students needed to use the hospital department plan, teaching aids, etc., to conduct an interactive discussion and engage in emergency decision-making and on-site actions instead of running the case. Every simulation study carried out in the laboratory included three steps: 1. Orientation, including an introduction to the learning objectives and the operating environment of the case. 2. Simulation, where student ran the cases. 3.Debriefing, when the facilitators gave feedback to the students and summarised their performance in the laboratory.

### Semi-structured interviews

Qualitative research commonly uses semi-structured interviews, as these enable participants to reflect on past experiences and to generate new ideas on how to promote student engagement as they engage in retrospection. This process can shed light on useful and effective learning strategies. The main interview questions include the following: (1) How does student engagement and participation unfold in a simulation-based learning environment? What are the characteristics of that engagement and participation? (2) What are the differences between student engagement in the simulation-based learning environment and learning in traditional classrooms? (3) To what extent do you think you have invested more in student engagement in a simulation-based learning environment? The concrete content is shown in Interview Guide. After obtaining consent, we conducted the interviews. When collecting data, we would engage in “epoche” and suspend our previous theoretical views, allowing participants to express their personal feelings without restriction, and we tried to help them to speak to as many real and comprehensive feelings and different experiences as possible to obtain richer themes and a more comprehensive display of the different types and characteristics of student engagement.

During the interviews, the researchers conducted in-depth exploration, mainly around these open research questions, but always allowed the interviewees to fully express their thoughts without interference. Follow-up questions and investigative questions were asked if needed. Follow-up questions were used when researchers were very interested in the views the interviewee was stating and wanted to have a deeper understanding, in which case the researchers asked the interviewee to give more detailed information. Investigative questions are a technique that allows the discussion to continue while obtaining further clarification, which helped us manage the conversation with the interviewee so that it always revolved around the main research questions. We used some simple phrases, for example, “You did a good job, please continue”, to suggest the discourse depth we needed from the interviewees or to encourage them to further expand the conversation. All researchers used almost the same main questions and interview techniques for the 10 interviews, and interviewees could fully express their views about these broad questions. However, the researchers also reflected upon the interview recording after each interview with 2 students to better guide the next interview.

### Participants

This study used the principle of purposeful sampling, i.e., non-probability sampling, to select interviewees that would allow them to draw the maximum amount of information for the research questions in light of the research purpose [26]. We followed the following strategies: (1) participants were selected who could provide essential and sufficient information regarding the research questions to enhance the intensity sampling principle. That is, in the current study, priority was given to participants who were interested in the research topic, good at expression, open-minded and willing to share their experiences with others. (2) Adhering to the maximum difference sampling strategy [26], the results should reflect the heterogeneity within various learning experiences to the greatest extent, accounting for factors such as majors and sex that have proven to influence student engagement. First, according to the above sampling strategies, after communication with their instructors, we chose students who were active, highly engaged, and good at sharing in the classroom to be our interviewees. We selected a total of 10 students from different majors, adopting the snowball sampling method, who were recommended by the interviewees. They were all sophomores at a medical university in China. The descriptive information is shown in Table 1. Our choice was based on China’s medical education context. In China, there are many independently established medical universities that recruit students majoring in clinical medicine, nursing, preventive medicine, and rehabilitative medicine. In the management of medical education at the national level, medical education-related documents are issued by the state, and clinical medicine, public health, rehabilitation, nursing and other majors are included in the scope of medical education for unified management. The students were numbered according to the initials of their professional names and sorted by number.

**Table 1:** Basic information and numbers of the students interviewed

Serial Number	Profession	Grade	Sex	Age range	Numbering
1	Nursing	Two	Male	18 ~ 21	H1
2	Clinical medicine	Two	Female	18 ~ 21	L1
3	Preventive medicine	Two	Female	18 ~ 21	Y1
4	Nursing	Two	Female	18 ~ 21	H2
5	Health Care Management	Two	Male	18 ~ 21	W1
6	Nursing	Two	Female	18 ~ 21	H3
7	Clinical medicine	Two	Male	18 ~ 21	L2
8	Imaging Science	Two	Female	18 ~ 21	Y2
9	Health Care Management	Two	Female	18 ~ 21	W2
10	Rehabilitation medicine	Two	Male	18 ~ 21	K1

### Data analysis procedure

This paper was informed by an approach based on thematic analysis [27]. Data collection and analysis were carried out simultaneously; that is, we conducted the data analysis immediately after interviewing 2 participants. The determination of the specific number of interviewees generally followed the “theoretical saturation principle”: when the newly extracted information about the types and characteristics of the interviewees’ learning engagement no longer contributed to new coding, additional interviews would not be carried out. All the interviews were audio recorded. In the process of thematic analysis, our own theoretical views on student engagement in a simulated learning environment provided the basis for in-depth dialogue with the materials. This study conducted interviews in Chinese, taking into account that the use of one’s native language allows the participants to more accurately reflect their ideas, and it follows the trend in which native languages are more frequently being used in qualitative research [28]. Two authors of this article, the first author majoring in English at the undergraduate level, translated the article. Additionally, three scholars were invited to conduct a strict review of the translation, two of which had studied and obtained doctoral degrees in English-speaking countries, and the other held a master’s degree in English. This process ensured a professional translation wherein translation errors would not affect the research results. We emailed the completed transcription to the participants, and they verified the transcribed text and redacted any information that they did not want used in the current study. We coded and analysed each text to find any content related to the research questions and identified the concepts and topics associated with the research interest. Before the analysis, the two authors discussed the theoretical framework, reached an agreement, and then analysed the transcripts independently. After the independent analysis, they resolved coding discrepancies and chose more appropriate coding and themes. The texts were organised and analysed by MAXQDA qualitative data analysis software version 10, with meaningful words, phrases, sentences, and paragraphs marked with a specific code. After the text data were coded, the codes were classified according to the theme. We then established a category and finalized the corresponding model framework. The final generic list is shown in Table 2.

**Table 2:** List of medical students’ learning categories

Secondary Coding Classification	Create A Generic	Learn Engagement Type
Focused, very focused Whole heartedly involved Active thinking Comprehend Find problems, analyze problems, solve problems Observe and listen Respond Record, write and reflect;	Concentrate on Concentration Mind and Body Involvement Active Thinking Problem Found Analyze Problem Solve the Problem Watch and Listen Submit Questions Express Opinions Record Reflection	Reflective Engagement
Upset, excitement, tension, excitement, eagerness to try, expectation; enthusiasm, joy, happiness, joy, excitement, sense of accomplishment; Discomfort, inferiority, depression, disappointment, loss, condemnation, guilt, Relax, tense; unconvinced, regret; Emotional expression, full of emotions; Strong emotional expression and indifferent emotions; Play a role Leadership, assignment of tasks, core members; Actual operation; Show oneself Not absent during important times	Positive Emotion Negative Emotion Emotional Expression Emotional Filling Strong Mood Indifferent Cosplay Leadership Core Member Actual Operation Strict Attendance	Performance Engagement
Immersive performance Rescue senior simulators and interact with them Identify peer issues; Drive peers; Discuss, communicate and communicate within the profession; Peer awareness Peer collaboration Ask questions; Observer, questioner, helper, prompter; Teacher guidance Positive teacher feedback	As It Is Contextual Interaction Peer Correction Drive Companions Professional Communication Peer Awareness Peer Collaboration Ask Questions Identity Change Teacher Guidance Teacher Feedback	Interactive Engagement

## Results

The characteristics of student engagement in the simulation-based learning environment were summarised into three types: reflective engagement, performance engagement and interactive engagement. Furthermore, sub-themes were identified, as described below. Table 3 containing the themes, subthemes and sample quotes is shown below.

**Table 3:** List of themes, sub-themes and sample quotes

Themes	Sub-themes	Sample quotes
Reflective engagement with the purpose of problem solving and active thinking	active, persistent, and focused thinking engagement	Everyone knows what they want to do, what tasks they have to accomplish, and how they can cooperate with others. In this way, our way of thinking is dedicated towards what we take the initiative to think about, what we want to do. We also take the initiative to complete our own tasks, start proactive communication and cooperation with others. In this sense, everyone’s way of thinking has become more active.
	self-directed-learning thinking engagement with the purpose of problem solving	I feel more self-driven to solve the problems if they come up. I do not rely on facilitators to guide me to solve the problem. After all, I cannot just leave the problem there without doing anything … what is more important is the way of thinking. That is, how to find problems and how to find better ways to solve problems.
	active “voice” in class	When the student listens to the facilitator very carefully and asks questions; or when analysing the case, the student can politely interrupt others in a timely manner, and give their own opinion... especially when the questions they ask are constructive, or if they really help others to think more about the questions.
Performance engagement characterised by strong emotional expression and leadership	strong emotional experience and disclosure	When we perform a live simulation in the laboratory, first of all, there is a sense of tension. You have to complete the tasks given by the facilitator. You don’t know if you can do it well. Second, when we engage in this work, we can easily reveal our emotions regarding whether the work progresses well, whether the result is good, and what has happened in the process. For example, if something goes wrong or when we make a mistake, we will be very anxious, there may be a sense of shyness and even shame. In short, our moods are fully revealed.
	demonstration of professional leadership	When the leader is directing the group, the leader will do their job meticulously. This includes how many people should be allocated to every activity and what every member should do... when the leader organises everything very thoroughly, I can tell that the leader is very engaged at that time.
Interactive engagement as a result of multidimensional interactions between learners, learning communities and the environment	interaction with realistic learning situations	Children can survive by double-compression. I feel that I have to do a good job... and then I would carefully read the materials given by the facilitator and then delve deeper into the textbooks. I want to successfully rescue the baby... because the baby would cry despairingly, that would make the atmosphere very intense... and then the sense of urgency in that situation is much stronger than the pressure imposed by facilitators or exams in reality.
	support from teammates	In order to complete a group task, we need mutual encouragement and support. I feel that someone is listening and someone is willing to respond to you. This sense of mutuality is very fulfilling and very encouraging. During interaction, I can feel the kind of enthusiasm of the classmates. They are motivated to do the task because they really value the patient and imagine what if the same experience should happen to them.
	collegial facilitator-student interaction	The facilitator, as an observer and a questioner, is not an answerer, um, is a helper, a prompter. The facilitator is more easy-going. The sense of distance and barriers are eliminated.

### Reflective engagement with the purpose of problem solving and active thinking

#### Engaging in active, persistent, and focused thinking

One of the most significant features of cognitive performance in a simulation-based learning environment is “active reflective engagement”. Many participants in our study emphasised in the interviews that this is a kind of active thinking and active engagement. Different from the short-term thinking in traditional classes, the simulation-based learning environment requires constant and persistent thinking. Another frequently used word is “focus”. Students who were highly focused in a simulation-based learning environment were deemed to have a “strong and attractive energy field” [L1]

*“[In traditional classrooms] Sometimes students are asked some questions but given limited time for a response. Many times, the class continues without giving enough time for student’s reflection on the questions.”* [L1]

*“[In a simulation-based learning environment] If you don’t take time to think about it, the patient in front of you may die, and you will feel a strong sense of guilt ... if you don’t solve the problem, things won’t work out. I will try my best to do it, not to give up halfway ... unlike in a traditional classroom, when the facilitator asks a question, you just need to give a standard answer and it all ends.”* [H1]

The simulation-based learning environment also allows students to be more focused on listening to the facilitator. They feel that they must take the lesson seriously because the follow-up session contains real-life practice.

*“In traditional classrooms, sometimes the facilitator talks too fast, and you will feel a bit unwilling to listen ... In the simulation-based learning environment, there is very little possibility of being distracted, you can focus more on the facilitator, and you can take more notes.”* [W2]

### Self-directed-learning thinking engagement with the purpose of problem solving

Students must familiarise themselves with the knowledge before entering the simulation-based learning environment. This is different from the passive learning practices in traditional classrooms, where students just follow the facilitator’s thinking, being instead a re-understanding of the original learning process and outcomes. Students are able to experience the self-directed learning process of constructing self-knowledge through observation, reflection, practice, problem discovery and resolution. Problem solving is the driving force of thinking activities, and the thinking process is reflected in the process of problem solving.

*“I feel more self-driven to solve the problems if they come up. I do not rely on facilitators to guide me to solve the problem. After all, I cannot just leave the problem there without doing anything … what is more important is the way of thinking. That is, how to find problems and how to find better ways to solve problems.”* [H1]

Problem-solving reflective engagement is accompanied by close connections between knowledge. Because it solves real-life problems, the knowledge being connected is situated in a multidisciplinary context, and the voices of the patients are also being heard.

*“In a normal session of your major, you just passively accept theoretical knowledge. You would not evaluate doctor-patient relationship from multiple perspectives, such as the potential influence over society and a humanistic perspective. I think my way of thinking has become more active.”* [Y1]

The process of problem solving is usually accompanied by communication and mutual affirmation among peers. When questions and thinking are valued, students are more motivated to think actively.

*“If you have your own thinking and vocalize it, everyone will listen more carefully to your suggestions and then put forward their own views... your own thinking is built up or challenged by others, which pushes you to do more active thinking.”* [L1]

### Active “voice” in class

“Speaking” in the classroom is another important manifestation of reflective engagement. On the one hand, an individual has more opportunities to ask constructive questions than in the simulation environment. On the other hand, unlike the commonly seen shyness and silence among East Asians [29], in a simulation-based learning environment, students are more willing to actively engage in conservations and answer questions raised by peers.

*“I am more involved than usual in [simulation] class. In traditional classrooms, students just sit in class and dare not answer facilitators’ questions. And now [in simulation class], if your peer asks you questions, you can quickly give a response, since if no one knows the answer, the game will not continue. The facilitator usually asks questions in class. The class gets embarrassing if no one speaks ... [in simulation class] I feel the need to talk about it myself, whereas there is no such feeling in [traditional] class.”* [H2]

In regard to the reasons why students want to speak up more in a simulation class, many participants think that “because of the group-discussion format, the group activities ease the classroom tension, which makes you more relaxed and can stimulate your thinking.”[Y1].

### Performance engagement characterised by strong emotional expression and leadership

#### Strong emotional experience and disclosure

Learning in a simulation-based learning environment usually requires students to play different roles, such as doctors, nurses, patients, and family members. Through these exercises, the students are expected to be familiar with the different roles to achieve the learning goals. Therefore, they often claim to be students from “a certain performing university” or “a performance major”. The description “acting elf” is often used to refer to those who are particularly good at acting.

Performance means that it is necessary to channel emotions into the character and act out that character’s behaviours. The current study found that strong emotional experience and disclosure are an important manifestation of performance engagement.

*“As far as I observe, the extent of student participation is diverse. Some people more actively participate, while some individuals are indifferent to emotional performance if they don’t pay much attention to this course.”* [W1]

The emotions in the simulation-based learning environment change in accordance with the progress of problem resolution and are most often associated with the outcome of the patient. There could be “unconsciousness, excitement, tension, eagerness, expectation” when the situation of the “patient” is unknown; there could be “happy feeling, confidence, excitement and a sense of fulfilment” if the operation is successful; there could also be “regret, discomfort, guilt, inferiority, depression, depression and feeling lost, unconvinced or even condemned” when the operation fails.

Strong emotional engagement enables students to be physically and psychologically involved in learning activities, contributing to a state of “presence”.

*“That kind of atmosphere [in a simulation environment] is different. The physical environment and learning atmosphere affect my psychological state and make me more focused on being involved in the task. I do not feel like just physically being in a classroom while mentally distracted by something else.”* [H1]

Many students even enter a state of “flow” when they fully immerse themselves in the tasks.

*“In the laboratory, I don’t care too much about how long the course takes. I think even if this class overruns, I won’t be too bothered.” [H2]*

Emotions and feelings are attitudinal experiences stimulated by a sense of “need” and revealed through behavioural outcomes. Although emotions and feelings are inspired by objective experiences, their nature is determined by people’s perception of the situation [30]. During the interview, some participants compared emotions, describing them as more “calm” in traditional classrooms but “fuller” in the simulation-based learning environment. Compared with the traditional classroom atmosphere, which suppresses the expression of emotions, a more relaxing simulation-based learning environment helps release various emotions.

### Demonstration of professional leadership

The simulation-based learning environment usually creates a realistic hospital scene. In this atmosphere, there are many actions that reflect professional leadership. These include whether one can take the initiative to take responsibility as the leader, whether one can lead the team to facilitate the learning process, or whether one actively follows the leader, quickly adjusts to the role, analyses problems, and offers responses. A deeper level of participation is reflected when those “leaders” use their knowledge and demonstrate their capabilities. As some participants said,

*“Some people will take the responsibility of leadership voluntarily and spontaneously assign people to do certain things. I will consider that person very devoted and motivated. For those who follow the lead, I also think that they are thinking deeply and engaged entirely”.* [L1]

### Interactive engagement as a result of multidimensional interactions between learners, learning communities and the environment

#### Interaction with realistic learning situations

The most prominent feature of the interactive learning environment is the realistic working session, including mimicking the busy atmosphere as well as the vivid “doctor-patient” interactions in a physical hospital setup, which enable students to be fully drawn to the learning context.

*“The more realistic the environment is, the stronger feeling I have that similar situations would happen in real life practices.”* [K1]

*“At the time, the assessment involved a little baby [model] ... we played with him, and we felt a kind of attachment. We would say, ‘look at those babies’. There would be a kind of motherly love – a kind of feeling expressed spontaneously.”* [L1]

The professional responsibility of medical and healthcare students is also stimulated by the simulated environment, which fully brings medical and healthcare students into their professions.

*“For example, if you rescue the senior simulator successfully, it will give out some responses. For example, during the rescue, the student can monitor the “patient’s” pulse, blood pressure etc. ... in that simulated context, because of the role you play, you will develop a strong sense of responsibility and want to do it well.”* [H2]

*“I will subconsciously think that I am a professional and I can do this well. Then, I will not be distracted by others, since I just need to do the operation based on what I have learnt. Building on that, I will also think about how I can better use the knowledge.”* [H2]

This sense of professional responsibility also motivates students to achieve a higher level of engagement.

*“I'm probably more engaged at that time [in the simulated learning environment] than I would be if I were alone. That is, when someone wants to interact with you, it makes me feel that this is not my own business. It is not like normal practice, which is solely operated by a single student, which easily makes one lonely and bored. Because there are interactions in a strongly collaborative atmosphere, which promotes my sense of responsibility. That is, if I do it wrong, my teammates will have to bear the consequences too, so it is not my own business.*” [H3]

#### Support from teammates

Peer interaction is mainly manifested in the sharing of information, mutual discussion, and skill guidance required in the process of problem solving.

*“You exchange views with your peer... and jointly solve this problem, which leads to increasing peer support.”* [K1]

Unlike ordinary peer communication, in the simulation-based learning environment, peers are more conscious and conduct more in-depth communication within the profession and develop the ability to think about problems holistically.

*“What are their [my peers’] concerns? What are my concerns? What are the similarities and differences between my peers’ and my own concerns?”* [H1]

The “companion” in the simulation-based learning environment is not simply a learning partner but a buddy who needs to complete a task that is very challenging. The mutually facilitative interaction between peers promotes active engagement. However, if the interaction is not harmonious, it will hinder further engagement. Attentiveness and responsiveness from peers will drive the next round of positive emotional experience and student engagement.

*“If everyone is very cooperative and actively participates, the interactions will be smoother, it will have a more positive effect on participation. If everyone is cold and everyone does not want to talk, then this task cannot carry on.”* [H3]

The impressive performance of other students in the process of peer interaction also motivates the students to be more engaged.

*“The students who participated in that course are very active and full of positivity. I enjoy interacting with them, and I am willing to speak up if I feel like it... when my classmates are thinking about the problem very seriously and trying to solve the problem, I feel that I can’t stand by but need to help out.”* [K1]

*“Especially if your peers are very good at acting, they convince you that the entire process is very authentic and make you more engaged in solving the problems.”* [H2]

#### The collegial facilitator-student interaction

The role of the facilitator in the simulation-based learning environment is more that of a guide, directing the students forward instead of just giving them answers. Furthermore, they provide students with ample space for thinking. The facilitator also takes on the role of “director”, setting up scripts and scenes, observing the actors’ performance, and giving guidance. “The facilitator, as an observer and a questioner, is not an answerer, um, is a helper, a prompter.” [H1] The facilitator-student interaction in the simulation-based learning environment is more “easy-going” and “informal” than that in the traditional classroom.

*“The facilitator also participates. The facilitator and we students feel like friends. Unlike in the classroom, where one needs to be more respectful when asking the facilitator questions, there is no distinct boundary between the facilitator and students in a simulated learning environment.”* [L1]

*“The facilitator is more easy-going. The sense of distance and barriers are eliminated.”* [Y2]

The facilitator-student relationship in the simulation-based learning environment is based on a more equal footing, as there is no hierarchical relationship during knowledge transfer. Instead, the facilitator acts as a consultant. If students are given positive feedback and affirmation in a timely manner, they will be more willing to participate.

*“That is to say, the facilitator has high expectations for us. Thus, we must definitely treat the course seriously and put in more effort so as not to disappoint the facilitator.”* [W1]

## Discussion

This research is the first to study the feelings and experience of medical and healthcare students in a simulated learning environment from the perspective of student engagement. Based on the findings, it reclassifies the types of student engagement, forming a new student engagement type that fits the simulation-based learning environment and differs from the classic student engagement type. Our research divides student engagement into three dimensions: reflective engagement, performance engagement and interactive engagement. Compared with the dimensions of traditional learning engagement models, it fully considers performance engagement, which is a combination of thinking and physical engagement in a simulation-based learning environment, and the engagement generated by interpersonal interactions; these additions are innovative and show the characteristics of learning in a simulation-based teaching environment.

### Types and characteristics of medical and healthcare student engagement

This study provides insights into medical and healthcare student engagement in a simulation-based learning environment, finding that it involves reflective engagement, performance engagement, and interactive engagement. Reflective engagement is problem-oriented, with a focus on inducing active, persistent and focused thinking practices. It involves a deep, reflective and concrete processing activity. As D. H. Jonathan states, “learners learn from thinking, but not from technology” [31]. Even in a simulation-based learning environment using advanced technology, the fundamental principle is still to stimulate students’ persistent, active and focused reflective engagement. Reflective engagement is a primary type of student engagement among medical and healthcare students, as students use it to reflect upon and practice existing knowledge. A literature review also suggested that an increasing emphasis is being placed on reflection and reflective practice in medical curricula [32]. Through reflective engagement, students are able to fully utilise critical thinking to obtain an in-depth understanding, construct advanced knowledge to create a bridge between knowledge and experience, facilitate supportive peer interactions and reduce the amount of “silence” in classrooms.

The study also found a special form of student engagement in a simulation-based learning environment, namely, performance engagement, which is role-based emotional and behavioural engagement. It promotes empathetic experiences, facilitates interprofessional practice and encourages a stronger desire for leadership. Similar to findings in the research conducted by Ulrich et al., role play simulation allows participants to actually feel the emotions they might experience in the future and develop their professional values [33]. Emotional engagement is an important aspect of medical and healthcare student engagement. Cognitive scientists tell us that learning is a multilevel communication process at the cognitive, emotional and physiological levels. However, this study also found that, unlike the positive emotions in general student engagement, which positively affect learning outcomes [34], mixed emotions are experienced by medical and healthcare students in simulation-based learning environments. Many participants considered that, as long as the affective level was high, all emotions and feelings indicated engagement. The special need in medical practice to manage doctor-patient relationships and intense emotional responses makes the emotional development of students particularly important in medical and healthcare education. Medical and healthcare students need to develop the emotions of compassion, care and empathy, and to develop moral sensitivity and emotional regulation. These findings are in accordance with those on the value of emotional intelligence in medical and healthcare education, with increasing research evidence showing that doctors’ emotional intelligence can improve their ability to deliver safe and compassionate health care [35]. In this way, they can always manage the balance within an emotionally rich environment to avoid emotional burnout. Medical and healthcare educators need to understand how emotions develop and how technology facilitates emotional development. The simulation-based learning environment also creates a more vivid professional identity for students beyond the pure learner status. Students show group or individual leadership when they “act in the professional role”; this process confirms that the integration of technology into teaching helps promote students’ individual psychological functions from purely cognitive development to meaning acquisition and the dual development of identity.

Interactions within student engagement are reflected in the responses to the high-fidelity simulator, mutually supportive peer interactions and collegial facilitator-student interactions in a realistic simulated environment. The origin of learning is the real-world construction of environmental and social interaction. The application of emerging technologies such as virtual reality and augmented reality has gradually turned the simulation-based learning environment into an important learning space for medical and healthcare students [36]. In this space, peers, facilitators and students constantly construct knowledge through interactions and cooperation. Teamwork is crucial for success: varied backgrounds and interests enable students to solve the problems from different perspectives [37]. Eismann et al. showed that simulation courses have an influence on teamwork [38]. The current study found that the more closely the learning context is related to real-life practices, the more mutual encouragement takes place among peers along with more positive feedback from the facilitator, which in turn promotes student engagement. Similarly, as studied by Behrens et al., well-designed clinical scenarios, teamwork and feedback support students’ learning [39].

### Logical relationships between different types of student engagement

Medical and healthcare student engagement in a simulation-based learning environment promotes students’ whole-hearted focus. Specifically, reflective engagement facilitates deeper levels of critical thinking and knowledge construction; interactive engagement emerges from interactive activities in the learning space; and performance engagement gives rise to role-based group activities, promoting participation and reflection. These three forms of engagement are subject to the influence of many environmental and pre-existing factors. For instance, when students engage with the roles they play, they need to constantly reflect on the role’s behaviours, feelings and thoughts while modifying their acting based on their knowledge and previous experiences. Step-by-step practices and immediate stimuli in the learning environment prompt more reflections, encouraging students to continuously conceptualise and act upon their experience; at the same time, they facilitate the interaction between the character and the external environment, thereby forming a relationship framework. Medical and healthcare students’ reflective engagement, performance engagement and interactive engagement influence each other. The use of IT highlights the multidimensional interaction between individuals, groups and the technology environment.

The findings indicate two perspectives that explain the mechanisms behind student engagement: the two-way construction of individuality and space in learning and the interdependence of the learner and the learning community. Facilitators can improve their teaching in a simulation-based environment accordingly. First, in terms of the promotion of reflective engagement, expanding the learning space centring around “inquiry” helps strengthen reflective communication and dialogue and further facilitates the process of knowledge construction. Before the simulation teaching, the facilitator can use the learning situation analysis method to analyse the students’ existing knowledge base. In the orientation stage, facilitators help the students fully review the case-related knowledge, making students convert the knowledge into action when they operate the case. In the debriefing stage, facilitators guide students’ reflection, asking them to determine three to five drawbacks about themselves or their peers and provide answers. After class, facilitators assign reflective learning homework, such as asking students to complete a reflective diary to help them improve their reflective skills.

In terms of promoting performance engagement, facilitators need to set the stage with a relevant and realistic scenario [40]. First, it is necessary to write cases based on realistic clinical scenarios, for example, ensuring the patient’s symptoms and signs are in line with the development of the disease, real and challengeable. The plot helps students develop clinical reasoning ability. Second, it ensures that the environment of the simulation laboratory is close to that of the real clinical environment, such as the placement of instruments and equipment. Finally, it guides students on how to show real emotions when performing but also pays attention to the emotions shown by the other characters to that they can display empathy. Facilitators should encourage exchanges and interactions between students of different majors, thereby promoting the establishment and development of professional responsibility and professional leadership. Interprofessional education research evidence provides a clear indication that it can improve collaborative attitudes/perceptions and the knowledge/skills necessary for collaborative practice [41].

In terms of promoting interactive engagement, the role of the facilitator should be changed from imparting knowledge to guiding and helping. For example, when the case is not operating smoothly, the facilitator should intervene in a timely manner to promote the case. In the debriefing stage, the facilitator should guide students as they think about the learning goals that should be achieved and their current learning level, as well as the gap between the two. In promoting peer interaction, the accountability method requires students to take responsibility for their roles, and the self-evaluation and peer evaluation methods allow students to provide feedback on themselves and their peers, respectively, to promote peer-to-peer interaction.

### Limitations

The purpose of this research is to reflect on student engagement in a simulation-based learning environment from students’ perspective, offering a basis for reference by facilitators to improve their methods of instruction. However, this is also a limitation of this research, which fails to make judgements about students’ learning achievements in combination with their objective learning performance. Our follow-up research direction is to overcome this limitation. This research focuses on the learning experience and subjective feelings of students in the simulation laboratory. However, we cannot know their possible reactions in the real world, nor can we know whether the learning experience in the laboratory will affect their performance in the real world. This is also a possible future direction of our research.

## Conclusions

Student engagement in a simulation-based learning environment is different from that in the traditional classroom. The current study provides evidence for the unique nature of simulation teaching, which promotes reflective learning and immersion in a clinical environment while facilitating reflective engagement, performance engagement and interactive engagement. These three types of student engagement help them to achieve the expected learning result through the two-way construction of individuality and space in learning as well as through the interdependence between the learner and the learning community.

## Supplementary Information

**Additional file 1.**

## Data Availability

As these data are semi-structured interviews, raw data will not be made available to protect participant confidentiality.

## Abbreviations

TEL: Technology-enhanced learning
IT: Information technology.
