# Supplementary material for: How do they learn: types and characteristics of medical and healthcare student engagement in a simulation-based learning environment
Source: BMC Med Educ. 2021 Aug 6;21:420. doi: 10.1186/s12909-021-02858-7 (PMC8349045; doi:10.1186/s12909-021-02858-7)
Supplement: Supplementary file 1 — Additional file 1. [file 12909_2021_2858_MOESM1_ESM.docx]

**Interview Guide for medical and healthcare student engagement in the simulation-based learning environment**

Dear students,

The aim of this interview is mainly to understand student engagement in a simulation-based learning environment. Your answers are very important for our subsequent medical education reforms. Thank you for your participation. The answers will only be used in the related journal articles and project reports. Any data that may be traced back to your personal information will be treated anonymously, and your personal information will not be disclosed. The interview will last approximately 40 minutes to an hour.

The main questions of the interview include:

(1) How does student engagement and participation unfold in a simulation-based learning environment? What are the characteristics of that engagement and participation? (2) What are the differences between student engagement in the simulation-based learning environment and learning in traditional classrooms? What changes have occurred in your learning style? In what ways have you been more involved in the learning? (3) To what extent do you think you have invested more in student engagement in a simulation-based learning environment?

School of Nursing, Nanjing Medical University
